# Spatial reconstruction feature for object detection on coal mine

**DOI:** 10.3389/frai.2026.1959087

**Published:** 2026-10-28

**Authors:** Haoxuanye Ji, Pengfei Jiang, Ziyue Wang, Zhiliang Chen, Yijie Liu

**Affiliations:** 1 China Coal Technology & Engineering Group (CCTEG) Coal Mining Research Institute Co., Ltd., Beijing, China; 2 Tiandi Science and Technology Company Ltd., Beijing, China; 3 China Coal Technology & Engineering Group (CCTEG) Intelligent Strata Control Technology (Tianjin) Company Ltd., Tianjin, China

**Keywords:** coal mine workface scenarios, feature pyramid, object detection, spatial reconstruction features, super-resolution reconstruction loss

## Abstract

Object detection has extensive application demands in coal mine operation scenarios. However, conducting high-quality object detection under complex underground backgrounds and harsh environmental conditions remains challenging. Most existing object detection methods adopt feature pyramids to retain the spatial and semantic information of the objects. However, it remains a critical challenge to extract features that preserve detailed object information from degraded images of coal mine workfaces. To address this issue, we propose a spatial reconstruction feature generation method for object detection from underground coal mines. The method first upsamples the feature pyramid via transposed convolutions to generate spatial reconstruction features. Subsequently, to ensure the effectiveness of the generated spatial reconstruction features, the stepwise super-resolution feature reconstruction loss supervises the feature pyramid layer by layer, introducing key target information from the image into the spatial reconstruction feature pyramid to assist the target detection process. The results in the underground coal mine target detection dataset DSLMF+ show that after introducing spatial reconstruction features, the mAP@50 of the YOLO-v5 object detection framework can be increased by an average of 1.65%, and the mAP@50 of the DETA object detection framework can be increased by an average of 2.42%. These experimental results demonstrate the effectiveness of the proposed method.

## 1 Introduction

Object detection methods have been widely applied across coal mine workspaces, including excavation, mining, and transportation, to enhance personnel safety, positioning, and localization of miner operations (Yang et al., 2023; Cheng et al., 2022). Although object detection algorithms have advanced significantly in recent years for conventional scenarios (natural lighting), complex underground coal mine environments still pose substantial challenges. These include image quality degradation from non-uniform lighting, dust, and other factors, as well as complex scenes and high demands for detecting small objects accurately. Therefore, enabling algorithms to adapt to these challenges remains a focal point in research on applying computer vision algorithms to underground coal mine workface scenarios.

Object detection methods (Khanam and Hussain, 2024; Tomassini and Dewan, 2024; Lin et al., 2017) based on feature pyramids have achieved promising results for small objects. This is because feature pyramids retain feature information from small objects, preventing down-sampling operations such as pooling from erasing these features. However, even with preserved feature pyramids, performance is unlikely to be satisfactory in the complex background of the underground coal mine workface. This is because complex background features or non-object features can interfere with the discriminative features of small objects, as shown in Figure 1. To address these issues, Sun et al. proposed PiSA-SR Sun et al. (2025), which learns two LoRA modules on the Stable Diffusion (SD) model and introduces two adjustable guidance scales during inference to control pixel-level fidelity and semantic-level detail intensity, respectively. ISDNet (Guo et al., 2022) integrates infrared small object detection with object shape reconstruction to balance segmentation accuracy, memory usage, and inference speed. SuperYOLO (Zhang et al., 2022) fuses multimodal data and, through super-resolution learning, achieves high-resolution detection of multi-scale objects from low-resolution inputs.

**Figure 1:**
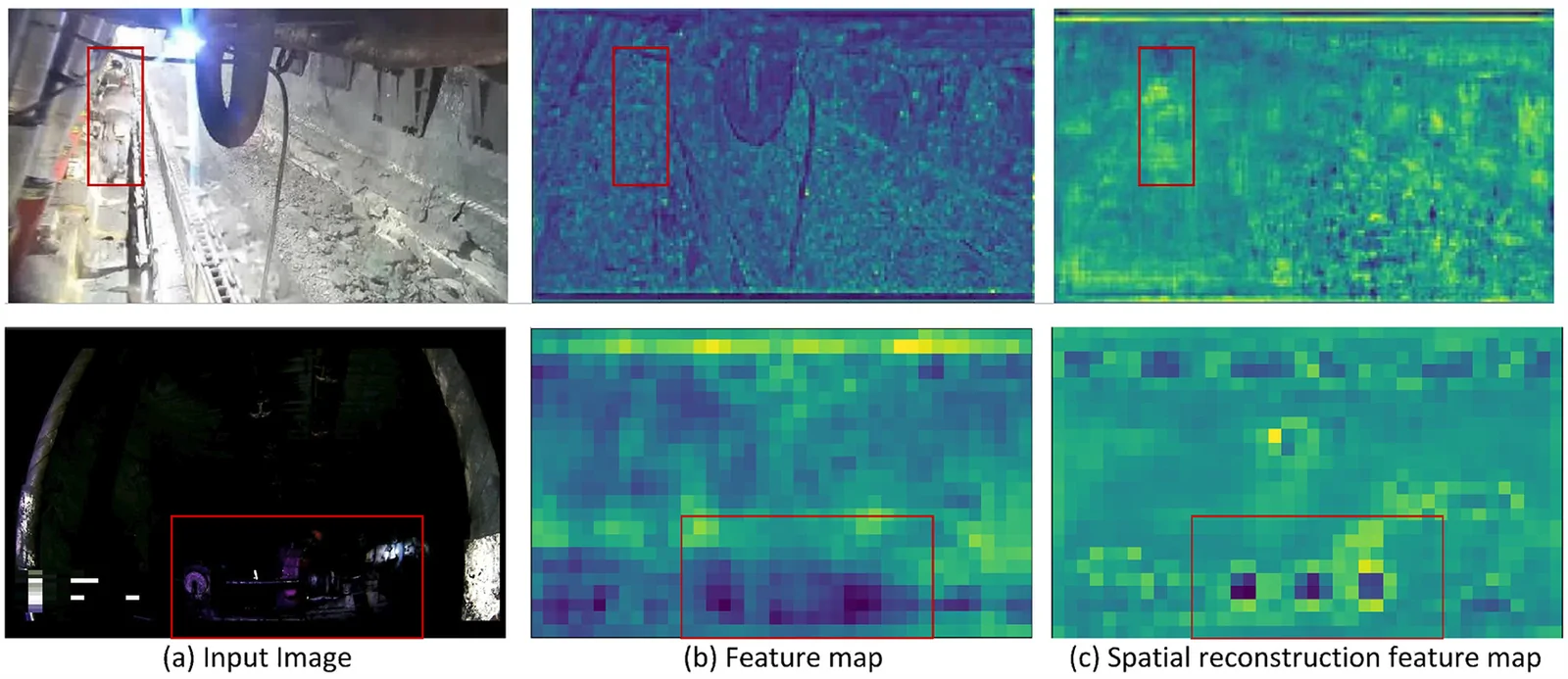
Illustration of the spatial reconstruction feature on the DSLMF+ “Coal miner” and the CUMDS dataset. **(a)** Original input image. **(b)** Feature map without spatial reconstruction. **(c)** Feature map with spatial reconstruction. The red bounding box indicates the object region, where spatial reconstruction features can encompass more characteristics of the object in the region.

Inspired by these methods, this paper introduces a spatial reconstruction feature generation module that integrates super-resolution information into the object detection task for underground coal mines. By employing a stepwise super-resolution feature reconstruction loss within the feature pyramid, the module effectively learns features specific to small objects, as shown in Figure 1c. Specifically, for an input image *x*_*i*_, the encoder first processes *x*_*i*_ into a set of feature pyramid representations. A set of designed learnable upsampling modules then upsamples these feature pyramid representations to the same size. To ensure the upsampled features contain sufficient, effective super-resolution information, we introduce stepwise super-resolution feature reconstruction losses. These losses supervise features from different pyramid levels using *x*_*i*_, preserving key super-resolution information in each layer and forming spatial reconstruction features. These features comprehensively capture the critical feature information of objects, especially small ones. In YOLO-based frameworks, these spatial reconstruction features can be used directly as queries for object detection in underground coal mines. For ViT-based frameworks, the object-related information from the spatial reconstruction features is fused into learnable query embeddings through a mutual attention mechanism, thereby enhancing the detection capability for objects, particularly the small ones, in underground coal mine environments.

The contributions of the paper are summarized as follows:

- This paper proposes a novel spatial reconstruction feature generation method for object detection in coal mines. By integrating spatial distribution information from the samples into the feature pyramid, it helps the features capture more key object characteristics, ensuring effective detection.
- This paper proposes a ladder super-resolution feature reconstruction loss that supervises each feature pyramid to ensure the spatial distribution information in the spatial reconstruction features is valid.
- This paper combines the proposed spatial reconstruction feature with YOLO- and ViT-based frameworks, demonstrating its effectiveness.

The remainder of this paper is structured as follows: Section 2 offers a concise summary of pertinent research. Section 3 introduces the proposed method, which leverages spatial reconstruction features for object detection in coal mine scenes. Section 4 presents experimental results and discussion to validate the proposed method's efficacy. Lastly, Section 5 presents the conclusion.

## 2 Related work

**YOLO detection methods** adopt an end-to-end framework that transforms object detection into a regression problem, directly predicting object bounding boxes and class probabilities from pixel-level features of the image (Tomassini and Dewan, 2024). YOLO method (Redmon et al., 2015) first proposed a one-stage object detection strategy and implemented a one-stage object detection framework based on GoogleNet (Szegedy et al., 2015). The YOLO-v2 method (Redmon and Farhadi, 2017) introduced the Darknet-19 backbone network, along with Anchor Boxes, batch normalization, and other techniques, significantly improving recall and localization accuracy. YOLO-v3 (Redmon and Farhadi, 2018) uses the Darknet-53 backbone, introduces multi-scale prediction, and replaces Softmax with logistic regression for class prediction, improving detection for objects of varying scales. YOLO-v4 (Bochkovskiy et al., 2020) adopts CSPDarknet53 as the backbone network, incorporates SPP (He et al., 2015) and PANet structures, and integrates training strategies such as Mosaic data augmentation, CmBN, and self-adversarial training, achieving high-precision real-time detection on standard hardware. YOLO-v5 (Khanam and Hussain, 2024) incorporates the CSPNet concept and introduces Focus slicing operations and more flexible adaptive anchor box calculations, further optimizing speed and accuracy. YOLO-v8 (Varghese and M., 2024) adopts an Anchor-Free strategy, introduces a Decoupled Head, and uses Distribution Focal Loss (DFL) and CIoU as loss functions, simplifying the detection process. CLF-YOLOv8 (Wang et al., 2026) integrates the Cross-stage Lightweight Fusion strategy into the YOLO-v8 framework (Varghese and M., 2024) for real-time night maritime detection. Shi and Song (2024) present the Defog YOLO algorithm, which integrates an enhanced U-Net framework for visual defogging, specifically designed for road object detection in foggy conditions. Li et al. (2026) proposed a semi-supervised learning framework DTRB-Net, which integrates a dual-teacher architecture with an adaptive reliable bank to learn discriminative features. CSS-YOLO (Han and Zhang, 2026) integrates a cross-channel feature fusion module, multi-branch SENetV2 channel attention, and a spatially coupled convolution block to enhance multi-scale semantic consistency, channel discriminability, and local spatial modeling, making it suitable for small and occluded objects in complex natural scenes.

**Transformer detection methods** treat detection as a direct “set prediction” problem. DETR (Carion et al., 2020) eliminates manually designed components such as anchor boxes and non-maximum suppression (NMS) in traditional detection, directly using a set of learnable query embeddings for object bounding box prediction. Deformable DETR (Zhu et al., 2020) introduces a sparse deformable attention mechanism, significantly improving training efficiency and computational performance. DN-DETR (Li et al., 2024) and DINO (Zhang et al., 2023) incorporate denoising training strategies that markedly accelerate convergence and improve performance. ViT-Adapter (Chen et al., 2023) is an adaptation module that adds multi-scale feature extraction and spatial-prior capabilities to the standard Vision Transformer (ViT) (Dosovitskiy et al., 2021), enabling it to handle dense prediction tasks (such as object detection and segmentation) efficiently without modifying the backbone structure. DAB-DETR (Liu et al., 2023) is an improved DETR (Carion et al., 2020) object detection model that enhances training convergence speed and detection performance by using dynamically changing 4-dimensional anchor boxes (*x, y, w, h*) as queries for the Transformer decoder.

## 3 Methods

### 3.1 Overview

To address the issue that the characteristic information of small objects in the feature map is prone to being ignored, we propose a reconstruction feature generation module that reconstructs a super-resolution feature map. This module uses the designed super-resolution decoding method to upsample each level of the feature pyramid. Then, a ladder super-resolution reconstruction loss is adopted to fit the appearance differences between the upsampled features and the images. We focus mainly on the feature-coding part of the network, as shown in Figure 2.

**Figure 2:**
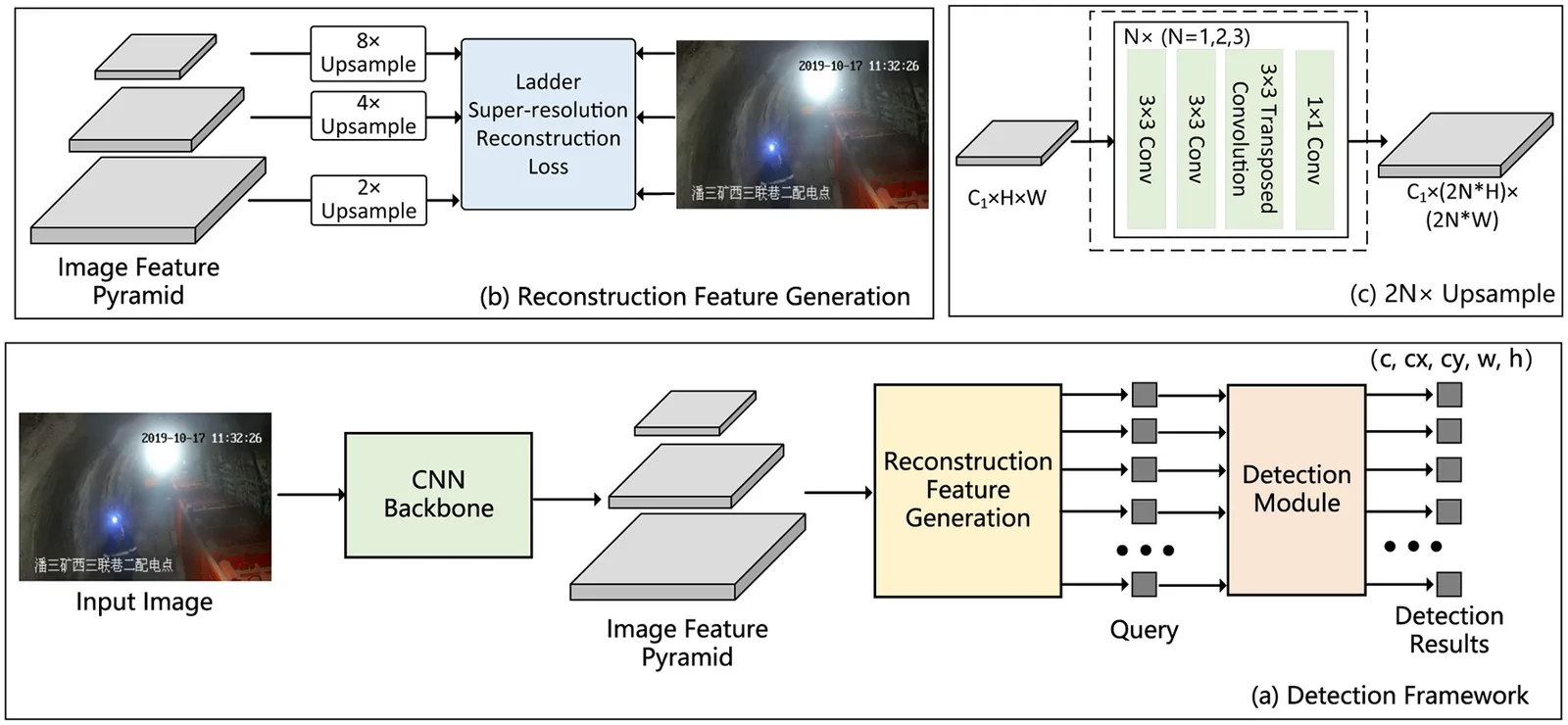
Object detection framework with our proposed reconstruction feature generation module. **(a)** Detection framework. **(b)** Reconstruction feature generation. **(c)** 2*N*× upsample.

During training, given an image and its corresponding bounding boxes, a Convolutional Neural Network (CNN) extracts a spatial feature pyramid. Then, a set of upsampling operations is used to reconstruct each feature in the spatial feature pyramid, and a designed ladder super-resolution Reconstruction Loss is used to measure the difference between the upsampled feature and the input image, thus training the query vectors for object detection. Furthermore, the deep neural network framework for object detection is trained through the L_1_ regression loss, object cross-entropy loss, and pixel-level mask classification loss between the predicted object bounding box and the real object bounding box.

In the inference stage, given an image with the spatial feature pyramid extracted by the CNN backbone. The introduced reconstruction feature generation module is to further enhance the described capability of features for object characteristic information. Subsequently, the generated features are used as query vectors in an object detection module, such as a YOLO-like or DETR-like framework, and the object detection results are obtained through classification scores and NMS.

### 3.2 Spatial reconstruction feature generation

The loss of object spatial characteristic information caused by CNN down-sampling operations (*e.g*., pooling) is the main reason for false detections or missed detections. Therefore, we consider sampling and reconstructing the multi-scale features in the feature pyramid and using these reconstructed features as query vectors for object detection. As shown in Figure 2b, the generation of the reconstructed features is divided into two steps, namely upsampling and learning the reconstructed features.

#### 3.2.1 Upsampling

Upsampling operations are used to unify the feature sizes of feature pyramids of different sizes to adapt to the learning of reconstructed features. As shown in Figure 2c, the operation includes two layers of 3 × 3 convolution to further increase the receptive field of the features, a layer of 3 × 3 transposed convolution upsamples features through learnable parameters, and this process can be formulated as shown in Equation 1:


$$\begin{array}{l} y_{2}={\text{ConvTrans}}_{3\times 3}\left({\text{Conv}}_{3\times 3}\left({\text{Conv}}_{3\times 3}\left(y_{1}\right)\right)\right), \end{array}$$ (1)

where $y_{1}\in {\mathbb{R}}^{C_{1}\times H_{1}\times W_{1}}$ means the input features, $y_{2}\in {\mathbb{R}}^{C_{1}\times \left(2*H_{1}\right)\times (2*W_{1}})$ is the output features after upsampling operation. Then, a 1 × 1 CNN layer is adopted to carry out the channel transformation, which is formulated as in Equation 2:


$$\begin{array}{l} y_{3}={\text{Conv}}_{1\times 1}\left(y_{2}\right), \end{array}$$ (2)

where, $y_{3}\in {\mathbb{R}}^{C_{2}\times \left(2*H_{1}\right)\times \left(2*W_{1}\right)}$ denotes the channels' number of *y*_2_ is transformed from *C*_1_ to *C*_2_. Alignment with the scale of the input image can be achieved through cyclic superimposition of upsampling operations.

#### 3.2.2 Learning reconstruction feature

After completing the upsampling of the image features, it is necessary to design a learning strategy to fit the feature distribution differences between the upsampled feature image and the real image, considering the spatial reconstruction and feature perception of the target objects in the image by the feature map. A high-quality fitting process requires making full use of the pixel spatial relationship in the upsampling feature (*i.e*., *y*_3_).

Specifically, the reconstruction feature *y*_3_ is used to construct the adjacency subgraph **G** = (**A**, **V**), where **A** represents the adjacency matrix, and **V** is the node features in the adjacency subgraph. Each pixel feature *p* in *y*_3_ constructs the adjacency matrix **A**, which is represented as in Equation 3:


$$\begin{array}{l} \text{A(a, b)}=\{\begin{array}{l} \text{1, }{\text{p}}_{\text{a}}\in {\text{NN}}_{\text{k}}\text{(}{\text{p}}_{\text{b}}\text{)}\\ \text{0},\text{ otherwise} \end{array}, \end{array}$$ (3)

where NN_*k*_(*p*_*b*_) denotes the *K*-Nearest Neighbor nodes of the pixel feature *p*_*b*_. The corresponding adjacency loss function can be described as in Equation 4:


$$\begin{array}{l} L_{\text{adj}}=\frac{\sum_{a=1}^{W}\sum_{b=1}^{H}{\text{E}}_{\text{a, b}}\cdot |y_{a,b}-{ŷ}_{a,b}|}{W\cdot H};\text{E}={\text{D}}^{-1}\cdot \left(\text{A}+\text{I}\right), \end{array}$$ (4)

where **D** represents the Laplace operator of **G**. **I** is the identity matrix. *y* represents the feature map of *y*_3_ after channel transformation. ŷ_*a,b*_ denotes the ground truth pixel value. Furthermore, the pixel loss $L_{\text{pixel}}$ promotes the overall accuracy of the reconstructed image; that is, the pixel values are directly similar to the values of the original image. This ensures no significant changes in the image's color, lighting, or overall contrast. $L_{\text{pixel}}$ can be described as in Equation 5:


$$\begin{array}{l} L_{\text{pixel}}=\frac{\sum_{a=1}^{W}\sum_{b=1}^{H}|y_{a,b}-{ŷ}_{a,b}|}{W*H}. \end{array}$$ (5)

To sum up, the ladder super-resolution reconstruction loss can be formulated as in Equation 6:


$$\begin{array}{l} L_{\text{recon}}=\overset{N}{\underset{n=1}{\sum }}\lambda_{n}*\left(\alpha *L_{\text{pixel}}+\left(1-\alpha \right)*L_{\text{adj}}\right), \end{array}$$ (6)

*y*_*n*_ represents the output of the *N*-th upsampling module. λ_*n*_ is a hyperparameter that is the weight of each feature in the feature pyramid, and α is the weight between $L_{\text{pixel}}$ and $L_{\text{adj}}$. By continuously training the proposed stepped super-resolution reconstruction loss, the feature map can be reconstructed iteratively and used in the detection process of small objects.

### 3.3 Object detector optimization with spatial reconstruction feature

After the feature reconstruction learning process by the Algorithm 1, the generated super-resolution reconstruction features contain feature information about effective objects. These features can be flexibly used in the anchorage-free object detection framework. To this end, we introduce the reconstructed features into the YOLO-like and DETR-like object detection frameworks, as follows:

#### 3.3.1 Yolo-based framework implementation

The Neck network of the Yolo-v5 framework adopts the structure of FPN-PAN. The FPN layer conveys strong semantic features from top to bottom, while the PAN tower conveys positioning features from bottom to top. Moreover, the feature fusion ability of the network is enhanced through the CSP2 structure, thereby generating the corresponding feature pyramid. For the input image $x_{i}\in {\mathbb{R}}^{3\times H\times W}$, the features, which are used for object detection, can be expressed respectively as: $y_{1}\in {\mathbb{R}}^{3\times \left(H/8\right)\times \left(W/8\right)}$, $y_{2}\in {\mathbb{R}}^{3\times \left(H/16\right)\times \left(W/16\right)}$, $y_{3}\in {\mathbb{R}}^{3\times \left(H/32\right)\times \left(W/32\right)}$. Thus, the corresponding *N*_1_, *N*_2_, and *N*_3_ in Figure 2 can be set as 2, 4, and 8 respectively. Finally, the corresponding features generated in the Reconstruction Feature Generation are used as the “anchor features” for the object detection. In training process, ŷ ∈ ℝ^3 × (*H*/2) × (*W*/2)^ in Equation 1 is *x*_*i*_ after interpolation down-sampling. The object detection framework with the Reconstruction Feature Generation can be formulated as in Equation 7:


$$\begin{array}{l} L_{\text{base}}+L_{\text{recon}}, \end{array}$$ (7)

where, $L_{\text{base}}$ denotes the loss function of Yolo-v5 framework.

In summary, by introducing the Reconstruction Feature Generation module after the Neck network shared by the YOLO-like framework, we generate features with valid discriminative information for the object detection task.

#### 3.3.2 ViT-based framework implementation

The ViT-based framework utilizes the attention module in the transformer, which implements the object detection process by paying attention to the object information in each feature map. The ViT framework contains a ViT-based encoder and a decoder. By initializing a set of learnable query vectors and the key foreground features of the image produced by the encoder, accurate object detection results are observed. To introduce the spatial information of each image into the ViT-based framework, we introduce the spatial reconstruction features into the embedding encoder of the ViT-based framework, which is discussed in Section 4.5. By using the reconstructed features as the feature library of the input image, they are used in ViT-based encoders or ViT-based decoders to solve for the object positions contained in the input image.

**Algorithm 1:**
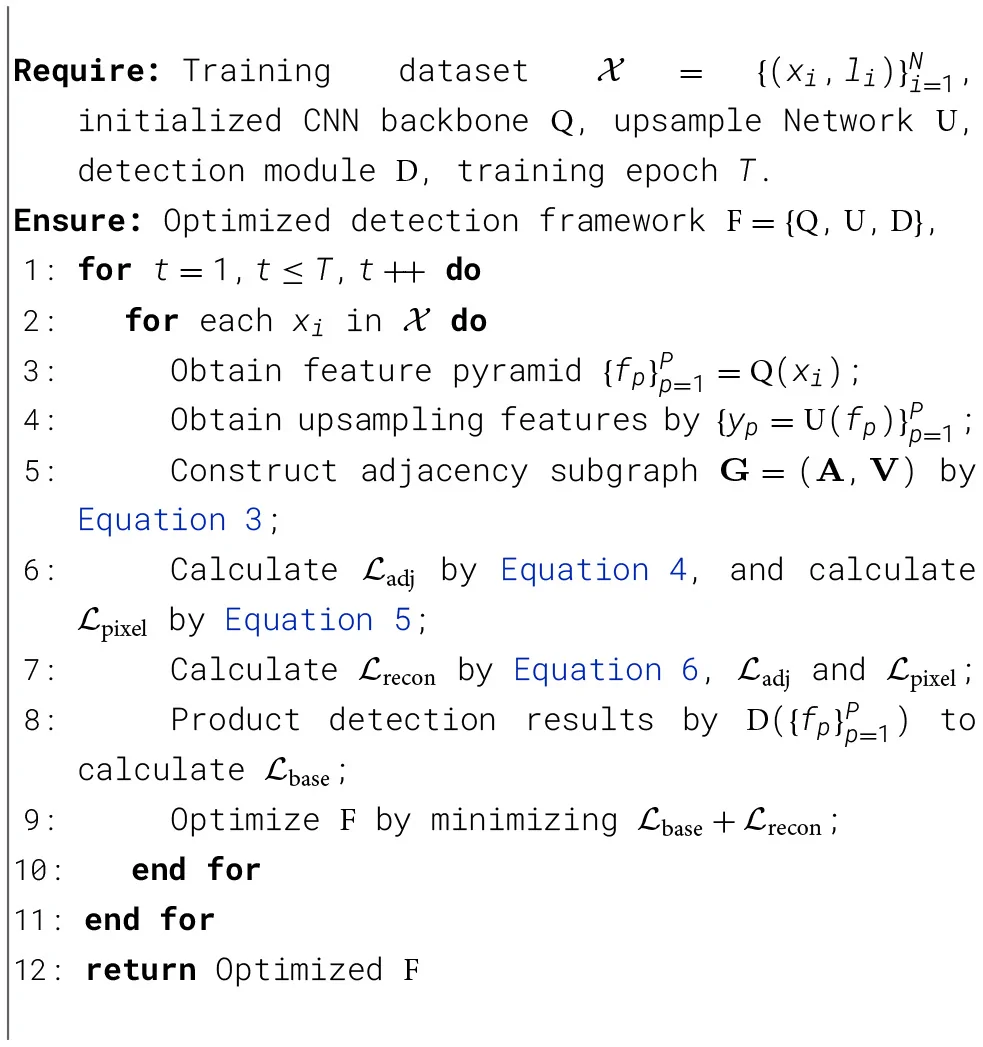
Optimization procedure.

## 4 Experiments and discussions

In this section, we evaluate the object detection performance of our method against state-of-the-art methods and carry out detailed ablation studies to isolate the performance contribution of each component.

### 4.1 Datasets and protocol

#### 4.1.1 Datasets

We evaluate the proposed method on six sub-datasets of the large-scale object detection dataset DSLMF+ (Yang et al., 2023) for an underground long-wall fully mechanized mining workspace.

- **DSLMF+** dataset (Yang et al., 2023) is an open dataset specifically designed for the intelligent recognition and classification of abnormal working conditions in underground long-wall mining faces. It contains 138,004 images, labeled with six types of objects: miners, hydraulic support guard plates, large coal blocks, trailing cables, miner behaviors, and mining safety helmets.
- **CUMDS** dataset (Wei et al., 2024) is an object detection dataset in a coal mine underground drilling site. It contains 70,948 images from different drilling sites and environmental background conditions, covering five categories of objects: gripper, chuck, coal miner, mine safety helmet, and drill pipe.

We compare our method with state-of-the-art methods on the DSLMF+ dataset, and conduct ablation studies on the DSLMF+ “Coal miners” and “Mine safety helmet” datasets.

#### 4.1.2 Evaluation protocol

Follow DSLMF+ (Yang et al., 2023), the AP_0.5_, AP_0.75_, and AP_0.5:0.95_ are used to evaluate the detection performance of the method. AP_0.5_ and AP_0.75_ mean the average precision (AP) with the intersection over union (IOU) between the prediction and the ground truth bounding boxes equal to or greater than 50% and 75%. AP_0.5:0.95_ denotes the average score of the AP with IOU ≥ 50% to IOU ≥ 95% of each 5%. Higher scores represent better performance.

### 4.2 Implementation details

We implement our method in PyTorch with two NVIDIA GeForce 4090 GPUs. We resize each training image to 640 × 640, then use random crop, random flip, and mosaic loading for data augmentation Khanam and Hussain (2024). To verify the effectiveness of the generated reconstruction features, we adopt YOLO-v5 (Khanam and Hussain, 2024) and YOLO-v7 (Wang et al., 2023) as the YOLO-based baseline framework, and utilize ViT-Adapter-L (Chen et al., 2023) and DETA (Ouyang-Zhang et al., 2022) as the ViT-based baseline framework. After incorporating the reconstruction features, the performance improvement reflects their effectiveness. The training epoch, learning rate (lr), and its lr-scheduler are the same as those of the corresponding baseline framework. We use the YOLOv5 (Khanam and Hussain, 2024) framework as the baseline to explore the contribution of each component of our method in the Ablation Study and Parameter Analysis. For the reconstruction feature generation module, we set *N* = 3 to ensure training speed. About $L_{\text{recon}}$, we set λ_1_ = λ_2_ = λ_3_ = 1/3 and α is setting as 0.3 to balance $L_{\text{pixel}}$ and $L_{\text{adj}}$, which are discussed in Parameter Analysis.

### 4.3 Parameter analysis

We explore the impacts of the feature weights λ_1_, λ_2_, λ_3_, and the balance parameter α in Equation 6 on the DSLMF+ “Coal miners,” “Mine safety helmet” and CUMDS datasets.

#### 4.3.1 Weights λ_1_, λ_2_, λ_3_

In Equation 6, λ_1_, λ_2_, λ_3_ present the weights of each feature in the feature pyramid, respectively. To avoid the complex grid search process, we set λ_1_ = λ_2_ = λ_3_ = λ. To explore the impact of λ in the experimental results, we select λ ∈ {1/15, 1/12, 1/9, 1/6, 1/3, 1}. The results are shown in Figure 3. **AP**_0.5_ and **AP**_0.5:0.95_ are both increased when λ < 1/3 and slightly decreased when λ > 1/3. The reason is that smaller λ values cause the generated reconstructed features to contain insufficient pixel-level information, while larger ones cause the task-specific features to drift slightly, thereby resulting in a slight decline in performance. Thus, we choose λ = 1/3.

**Figure 3:**
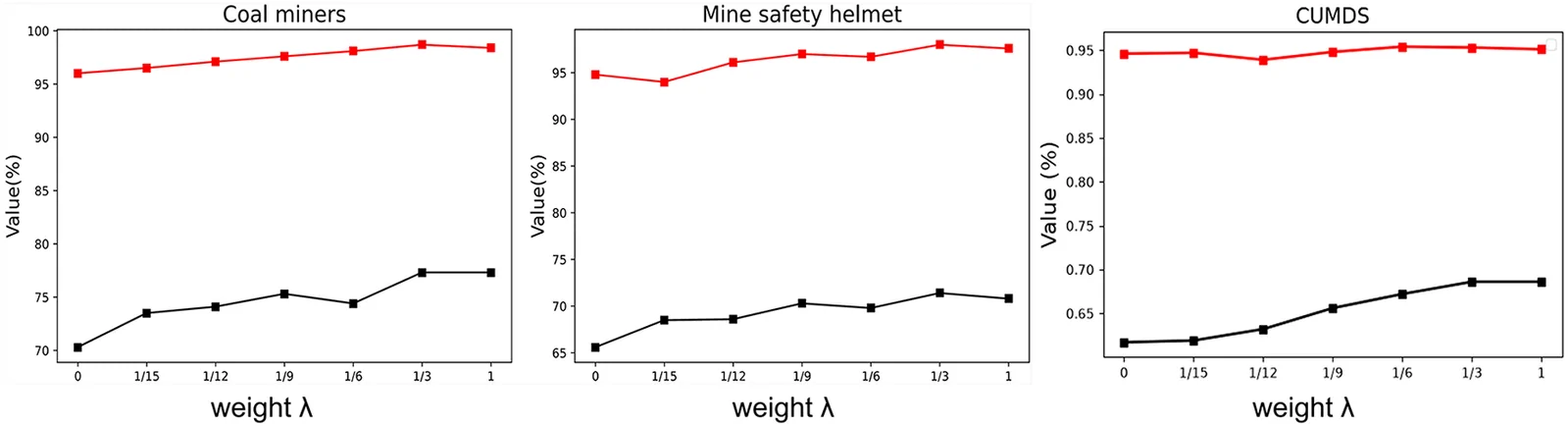
Experiments on the impact of the hyper-parameter λ in $L_{\text{recon}}$ on DsLMF+ “Coal miners,” “Mine safety helmet” and CUMDS datasets.

#### 4.3.2 Balance parameter α

In Equation 6, α is used to balance the loss function $L_{\text{p}ixe\text{l}}$ and $L_{\text{a}d\text{j}}$. To explore the impact of α, Table 1 records the numerical changes of *AP*_0.5_ and *AP*_0.5:0.95_ when alpha changes from 0.1 to 1.0 every 0.2. It can be seen that the performance is best at α = 0.5. Therefore, using appropriate features that can generate reliable spatial reconstruction features can better assist in the small object detection process.

**Table 1:** Experiments on the impact of the balance parameters α of generating reconstruction features on DsLMF+ “Coal miners,” “Mine safety helmet” and CUMDS datasets.

α	Coal miners		Mine safety helmet		CUMDS	
	AP_0.5_	AP_0.5:0.95_	AP_0.5_	AP_0.5:0.95_	AP_0.5_	AP_0.5:0.95_
α = 0	0.960	0.741	0.969	70.7	0.941	0.677
α = 0.1	0.956	0.737	0.977	0.699	0.937	0.673
α = 0.3	0.959	0.739	0.976	0.703	0.955	0.681
α = 0.5	**0.967**	**0.743**	0.980	**0.714**	**0.953**	**0.686**
α = 0.7	0.953	0.721	**0.981**	0.701	0.951	0.686
α = 1.0	0.949	0.722	0.968	0.686	0.947	0.662

Bold values indicate the best results among all compared methods.

### 4.4 Ablation study

We conduct a series of ablative experiments to evaluate the contribution of each module in our method.

(1) “Base.” selects the YOLO-v5 (Khanam and Hussain, 2024) as the baseline framework for object detection, the loss function $L_{\text{base}}$ is used for the framework learning process.

(2) “Base. + SRF ($L_{\text{pixel}}$)” includes a upsampling operation with the pixel loss $L_{\text{pixel}}$ to train the reconstruction feature for object detection.

(3) “Base. + SRF ($L_{\text{adj}}$)” incorporates the loss $L_{\text{adj}}$ to lead the feature reconstruction process for object detection.

(4) “Base. + SRF” is our full method, which incorporates upsampling operation, $L_{\text{pixel}}$, and $L_{\text{adj}}$.

#### 4.4.1 The effect of $L_{\text{pixel}}$

After incorporating the loss $L_{\text{pixel}}$ to optimize the reconstruction features, the AP_0.5:0.95_ is improved 3.1% on DSLMF+ (Coal miners) and AP_0.5_ and AP_0.5:0.95_ are improved 1.6% and 3.0% on DSLMF+ (Mine safety helmet) than “Base.”. This is because the reconstruction features include more pixel-level cues that prompt the anchor vectors for object detection.

#### 4.4.2 The effect of $L_{\text{adj}}$

After incorporating the adjacency information of the pixel features in each feature map in the training process, the AP_0.5_ and AP_0.5:0.95_ are improved 0.9% and 4.0% on DSLMF+ (Coal miners), and 2.1% and 5.1% on DSLMF+ (Mine safety helmet) than “Base.” Moreover, the AP_0.5_ and AP_0.5:0.95_ are improved 1.1% and 0.9% on DSLMF+ (Coal miners), and 0.5% and 2.1% on DSLMF+ (Mine safety helmet) than “Base. + RFG ($L_{\text{pixel}}$)”. Compared with the original learning, reducing the difference between pixel-level features allows adjacency cues to provide more useful pixel-level information about relevant characteristics for training.

To intuitively demonstrate the effectiveness of the $L_{\text{a}d\text{j}}$, we illustrate the heatmap of the SRF Figure 4a with the $L_{\text{a}d\text{j}}$ and Figure 4b without the $L_{\text{a}d\text{j}}$ in the training process, as shown in Figure 4. By leveraging neighborhood information, the discriminative power of each pixel feature with respect to target-related features can be enhanced. For instance, reflective strips on clothing can help detect miners, thereby strengthening pixel features' robustness to target-irrelevant features.

**Figure 4:**
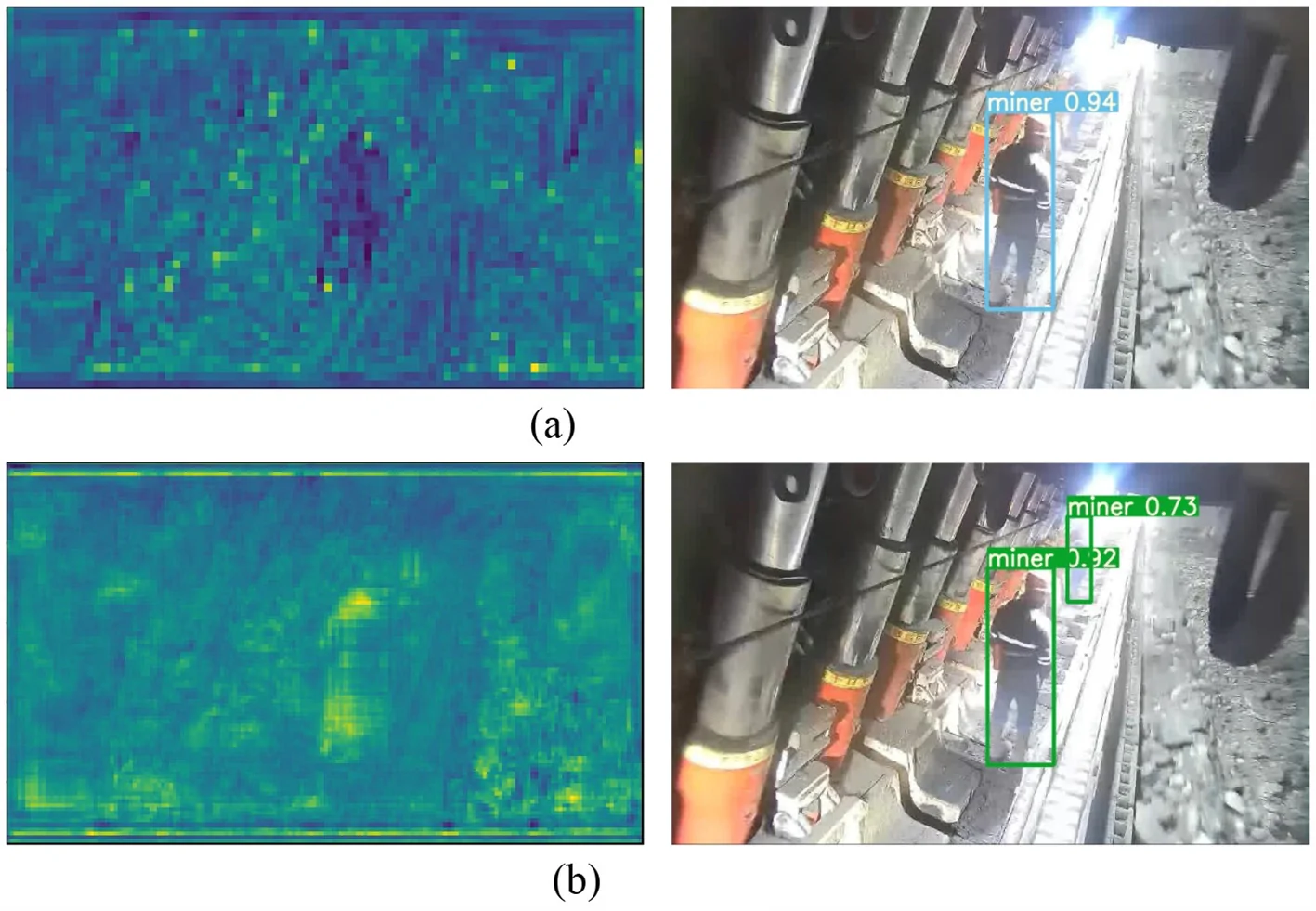
Some visualization results of the heatmap construction by the SRF optimization **(a)** without $L_{\text{a}d\text{j}}$ and **(b)** without $L_{\text{a}d\text{j}}$.

#### 4.4.3 Overall

“Base. + RFG” includes the above components, and the final performance is presented in the last row of Table 2. We could observe that AP_0.5_ and AP_0.5:0.95_ are improved by 1.6% and 4.2% on DSLMF+ (Coal miners) and 3.2% and 5.8% on DSLMF+ (Mine safety helmet) than “Base.”. In summary, our contributions significantly improve object detection performance.

**Table 2:** Ablation study on individual components of our method on DSLMF+ “Coal miners” and “Mine safety helmet” datasets.

Component	Coal miners		Mine safety helmet	
	AP_0.5_	AP_0.5:0.95_	AP_0.5_	AP_0.5:0.95_
Base.	95.1	70.1	94.8	65.6
Base. + SRF ($L_{\text{pixel}}$)	94.9	72.2	96.8	68.6
Base. + SRF ($L_{\text{adj}}$)	96.0	74.1	96.9	70.7
Base. + SRF	**96.7**	**74.3**	**98.0**	**71.4**

Bold values indicate the best results among all compared methods.

### 4.5 Comparison results

This section verifies the effectiveness of the proposed spatial reconstruction features in object detection tasks by comparing the performance of the SOTA methods and the spatial reconstruction features introduced into these methods in the large-scale object detection dataset DSLMF+.

#### 4.5.1 DSLMF+ dataset

The comparison results on DSLMF+ between our method and the state-of-the-art YOLO-like object detection methods are shown in Table 3. After incorporating the spatial reconstruction features in YOLO-v5, the mean AP_0.5_ and AP_0.5:0.95_ are increased by 1.65% and 2.03% on DSLMF+, respectively. About the YOLO-v7 framework, the spatial reconstruction features can improve the mean AP_0.5_ by 0.97% and AP_0.5:0.95_ by 3.85%.

**Table 3:** Performances of our method based on the YOLO-like frameworks on the DSLMF+ dataset.

Dataset		Yolo-V5	Yolo-V5-SRF	Yolo-v7	Yolo-v7-SRF
Coal miner	AP_0.5_	0.951	**0.967**	0.986	**0.990**
	AP_0.5:0.95_	0.701	**0.743**	0.773	**0.808**
Mine Safety Helmet	AP_0.5_	0.948	**0.980**	0.976	**0.984**
	AP_0.5:0.95_	0.656	**0.714**	0.679	**0.728**
Hydraulic support guard plate	AP_0.5_	0.961	**0.976**	0.978	**0.981**
	AP_0.5:0.95_	0.793	**0.818**	0.813	**0.841**
Large coal	AP_0.5_	0.828	**0.856**	0.868	**0.876**
	AP_0.5:0.95_	0.543	**0.598**	0.572	**0.639**
Miners' behaviors	AP_0.5_	0.915	**0.954**	0.913	**0.947**
	AP_0.5:0.95_	0.762	**0.778**	0.752	**0.777**
Towline	AP_0.5_	**0.990**	0.989	0.997	**0.998**
	AP_0.5:0.95_	0.907	**0.924**	0.916	**0.943**

Bold values indicate the best results among all compared methods.

The comparison results on DSLMF+ between our method and the state-of-the-art Transformer-like object detection methods are shown in Table 4. For the ViT-Adapter-L framework, we add a spatial reconstruction feature generation module to each Spatial Prior Module to enhance spatial information in the feature pyramid. The mean AP_0.5_ and AP_0.5:0.95_ are increased by 0.78% and 3.40% with the spatial reconstruction features. For the DETA framework, we adopt the DETR framework (Carion et al., 2020) and a one-to-many sample-matching strategy for object detection, rather than the one-to-one matching in DETR (Carion et al., 2020). Thus, we incorporate the spatial reconstruction feature generation module into the CNN backbone to enhance the spatial cues in the feature pyramid. Compared to “DETA,” the spatial reconstruction feature “DETA-SRF” can increase the mean AP_0.5_ from 93.53% to 95.95%, and AP_0.5:0.95_ from 67.37% to 71.70%.

**Table 4:** Performances of our method based on the Transformer-like frameworks on the DSLMF+ dataset.

Dataset		ViT-Adapter-L	ViT-Adapter-L-SRF	DETA	DETA-SRF
Coal miner	AP_0.5_	0.966	**0.971**	0.976	**0.989**
	AP_0.5:0.95_	0.702	**0.742**	0.684	**0.740**
Mine safety helmet	AP_0.5_	0.961	**0.970**	0.960	**0.976**
	AP_0.5:0.95_	0.624	**0.651**	0.601	**0.682**
Hydraulic support guard plate	AP_0.5_	0.963	**0.966**	0.958	**0.968**
	AP_0.5:0.95_	0.753	**0.770**	0.723	**0.742**
Large coal	AP_0.5_	0.854	**0.871**	0.815	**0.871**
	AP_0.5:0.95_	0.532	**0.581**	0.500	**0.537**
Miners' behaviors	AP_0.5_	0.928	**0.941**	0.914	**0.957**
	AP_0.5:0.95_	0.714	**0.767**	0.718	**0.753**
Towline	AP_0.5_	**0.989**	**0.989**	0.989	**0.996**
	AP_0.5:0.95_	0.871	**0.889**	0.816	**0.848**

Bold values indicate the best results among all compared methods.

#### 4.5.2 CUMDS dataset

The comparison on CUMDS between our SRF method and the state-of-the-art YOLO-like and Transformer-like frameworks is shown in Table 5. For the YOLO-like framework, SRF boosts mean AP_0.5_ and AP_0.5:0.95_ by 0.7% and 6.9% on YOLOv5, and by 0.5% and 5.4% on YOLOv7. About the Transformer-like framework, the mean AP_0.5_ and AP_0.5:0.95_ are improved by 1.8% and 4.8% on ViT-adapter-L after introducing the SRF. The mean AP_0.5:0.95_ is increased by 2.7% by DETA with SRF.

**Table 5:** Performances of our method based on the YOLO-like and Transformer-like frameworks on the CUMDS dataset.

Framework	mAP_0.5_	mAP_0.5:0.95_
Yolo-V5 Yolo-V5-SRF	0.946 **0.953**	0.617 **0.686**
Yolo-V7 Yolo-V7-SRF	0.949 **0.954**	0.604 **0.658**
ViT-Adapter-L ViT-Adapter-L-SRF	0.921 **0.939**	0.598 **0.646**
DETA DETA-SRF	**0.964** **0.964**	0.687 **0.714**

Bold values indicate the best results among all compared methods.

In summary, the results show that spatial reconstruction features can effectively boost detection performance, and we explain the gains from a technical perspective. First, the greater improvement in *AP*_0.5:0.95_ compared to*AP*_0.5_ across all frameworks indicates that the spatial reconstruction features primarily benefit precise object localization, because the stepwise super-resolution feature reconstruction loss forces the feature pyramid to retain fine-grained spatial details that are typically lost during downsampling, and these details are particularly important for achieving high IoU predictions under strict localization thresholds.

Second, the larger gains observed on CUMDS than on DSLMF+ (e.g., 6.9% vs. 2.03% for YOLO-v5 on *AP*_0.5:0.95_) suggest that the proposed features are more effective in degraded and complex environments, where images suffer from dust, low illumination, and motion blur that severely corrupt spatial cues; the spatial reconstruction features can compensate for these losses, thereby recovering discriminative information that would otherwise be inaccessible. Third, the consistent improvements across YOLO-like and Transformer-like frameworks demonstrate the generalizability of the proposed spatial reconstruction features: in YOLO-based detectors, the enhanced pixel-level features directly enrich the query embeddings used for object detection, whereas in ViT-based detectors, the inserted spatial cues improve the cross-attention alignment between object queries and feature maps, leading to more accurate classification and localization. Overall, these findings indicate that the spatial reconstruction feature is a flexible and effective plug-and-play component for both CNN-based and Transformer-based object detection frameworks.

#### 4.5.3 Computational overhead

We also discuss the parameters, FLOPs, and GPU memory of the YOLO-like^1^ and Transformer-like detection frameworks with a 640 × 640 image as the input image for the detection inference process, in Table 6. After incorporating the SRF, the parameters, FLOPs, and GPU memory are increased by 27.2*M*, 98.3*G*, and 0.606*GB*, respectively. These extra parameters are used to reconstruct features for introducing spatial characteristics information into the features.

**Table 6:** The parameters, FLOPs, and GPU memory of the YOLO-like and Transformer-like frameworks with the SRF Module.

Framework	Params.	FLOPs	GPU memory
Yolo-V5	86.7M	218.0G	1.6GB
Yolo-V5+SRF	113.9M	316.3G	2.2GB
Yolo-V7	154.7M	806.8G	6.0GB
Yolo-V7+SRF	181.9M	905.1G	6.6GB
ViT-adapter-L	347.7M	296.1G	3.5GB
ViT-adapter-L+SRF	374.9M	394.4G	4.1GB
DETA	210.0M	350.3G	7.8GB
DETA+SRF	237.2M	448.6G	9.3GB

### 4.6 Visualization results

To intuitively determine the effectiveness of our method, some detection results detected by YOLO-v5 and YOLO-v5+SRF on DSLMF+ “Coal miner” and the CUMDS dataset are shown in Figure 5. Image degradation and complex backgrounds cause objects to blend with the background clutter. Without SRF, YOLO-v5 struggles to distinguish objects from the background, leading to missed detections, as shown in Figure 5 (YOLO-v5). After incorporating SRF, the reconstructed object-pixel features enable the object detection model to better distinguish objects from the background, reducing missed detections, as illustrated in Figure 5 (YOLO-v5 + SRF).

**Figure 5:**
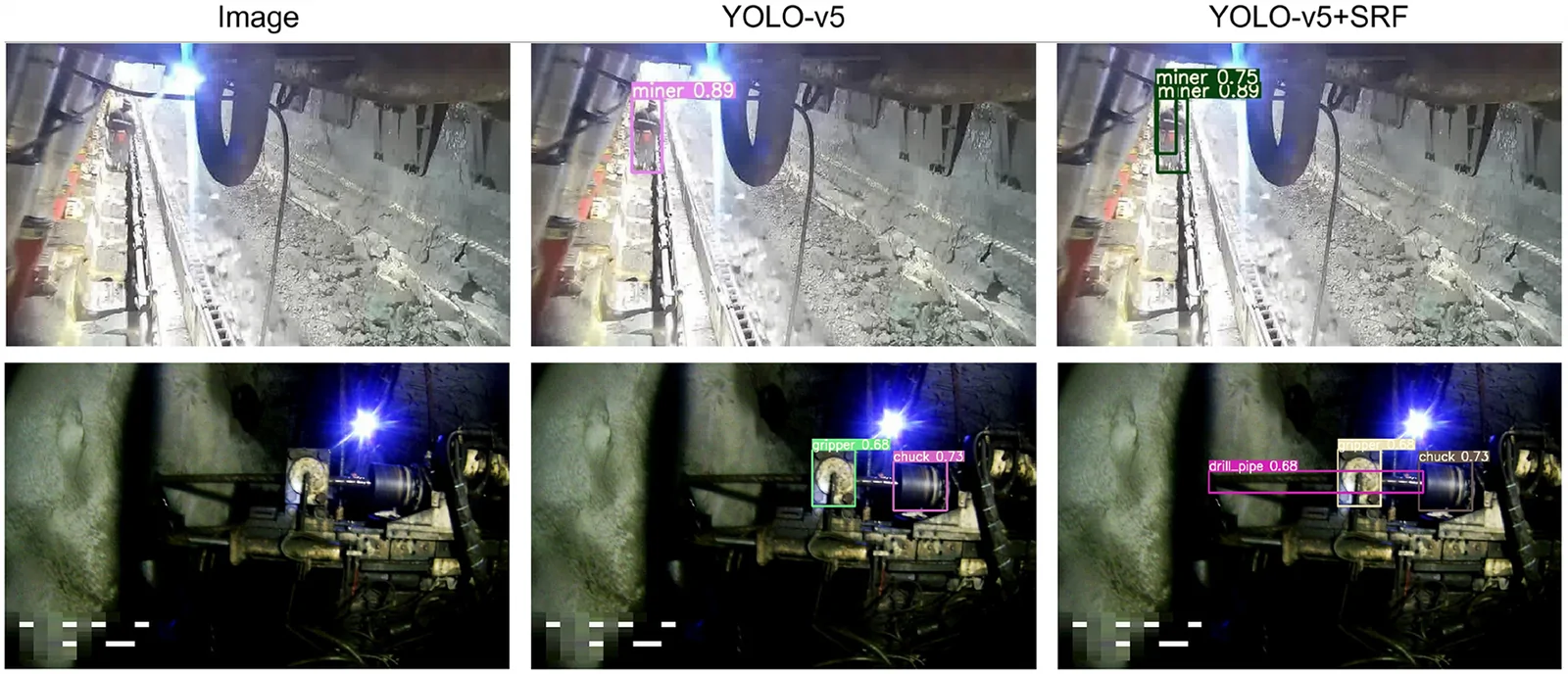
Visualization result on the DSLMF+ “Coal miner” and the CUMDS datasets.

## 5 Conclusion

This paper proposes a spatial reconstruction feature method for object detection in degraded coal mine images. It employs learnable up-sampling operations to restore each feature in the feature pyramid, generating the spatially reconstructed features. To ensure these features contain effective object information, the ladder super-resolution reconstruction loss is used to supervise the learning of inter-pixel relationships in the images in the training process. This enables the spatially reconstructed features to better characterize spatial distribution relationships within the image. Experimental results on the DSLMF+ dataset show that the spatially reconstructed features adapt to both YOLO-based and ViT-based frameworks, validating their effectiveness.

### 5.1 Limitations and future work

Degraded images from underground coal mine environments continue to adversely affect object detection performance; thus, the SRF includes more learnable parameters to reconstruct features and reduce these effects, which requires more computational resources. Future work will focus on integrating image denoising with object detection frameworks to develop an end-to-end approach for joint image denoising and object detection.

## Data Availability

The original contributions presented in the study are included in the article/Supplementary material, further inquiries can be directed to the corresponding author.

## Footnotes

1 Yolo-V5 and Yolo-V7 are the “x” version.
